# Tensions between real-world practices and the digitalization paradigm for data-driven services in eldercare: observations from an ethnographic study in Sweden

**DOI:** 10.1186/s12877-024-04693-z

**Published:** 2024-01-25

**Authors:** Mikaela Hellstrand, Maksims Kornevs, Jayanth Raghothama, Sebastiaan Meijer

**Affiliations:** Division of Health Informatics and Logistics, KTH Royal Institute of Technology, Hälsovägen 11C, Huddinge, Sweden

**Keywords:** Data driven health, Long term care, Quality of care, Relational care, Ethnography

## Abstract

**Background:**

The implementation of a data-driven approach within the health care system happens in a rapid pace; including in the eldercare sector. Within Swedish eldercare, data-driven health approach is not yet widely implemented. In the specific context of long-term care for older adults, quality of care is as much determined by how social care is being performed as it is by what kind medical care that is provided. In particular, relational aspects have been proven to have a crucial influence on the experience of quality of care for the actors involved. Drawing on ethnographic material collected at a Swedish nursing home, this paper explores in what way the relational aspects of care could potentially become affected by the increased use of a data-driven health approach.

**Methods:**

An ethnographic approach was adopted in order to investigate the daily care work at a long-term care facility as it unfolded. Fieldwork was conducted at a somatic ward in a Swedish long-term care facility over 4 months (86 h in total), utilizing the methods of participant observation, informal interviews and document analysis. The material was analyzed iteratively throughout the entire research process adopting thematic analysis.

**Results:**

Viewing our ethnographic material through an observational lense problematising the policy discourse around data-driven health approach, two propositions were developed. First, we propose that relational knowledge risk becoming less influential in shaping everyday care, when moving to a data-driven health approach. Second, we propose that quality of care risk becoming more directed on quality of medical care at the expense of quality of life.

**Conclusion:**

While the implementation of data-driven health approach within long-term care for older adults is not yet widespread, the general development within health care points towards a situation in which this will become reality. Our study highlights the importance of taking the relational aspects of care into consideration, both during the planning and implementation phase of this process. By doing this, the introduction of a data-driven health approach could serve to heighten the quality of care in a way which supports both quality of medical care and quality of life.

## Background

The implementation of a data-driven approach within the health care system happens in a rapid pace; including in the eldercare sector. In this (health care) context, data-driven approach (from here on conceptualized as *data-driven health approach*) means that health data is regarded as a valuable resource which, if handled in an appropriate way, can help to improve health care on a systemic level whilst maintaining or even heightening the quality of care as experienced by the individual patient [1, 2]. As part of the larger digitalization paradigm, policies sought to establish structures and procedures promoting a coordinated and integrated way of utilizing health data as a means of improving the quality and effectiveness of health care are being put forward and put into practice [3, 4]. In Sweden, this movement engages both the scientific community and health care sector, steered by ambitions set on a national-, as well as international (EU) level [2, 5, 6]. The health care sector is viewed as a complex system, a wicked problem [7], whose complexity is best tackled by using the most recent technological advancements for gathering clinical experience and knowledge in a cohesive way, making it accessible to stakeholders throughout the entire (complex) system [8]. Developments around precision medicine and preventative care make up two of the most emphasized aspects in connection to these two last points [9]. Further, a data-driven health approach is hypothesized to unleash the potential of utilizing a cohesive health data network to facilitate the collaboration and coordination between the different instances within the health care system [10, 11]. This is said to be extra relevant when it comes to older adult patients, as they are often in contact with several different health care providers during a prolonged period, often simultaneously.[12, 13]. The interconnection between health care and social care has been emphasized as particularly important [10]. What is further held forward for eldercare is the empowerment which older adults can get from having access to their health care records. This could provide older adults with the opportunity to take on a more active, kind of co-creating role in the planning of their care, e.g. by integrating their own self-tracking data and/or their self-perception on their health status into their health journals [14–16].

Much resources have been allocated for digitalizing Swedish eldercare, a measure which includes both the implementation of welfare technology as well as a re-organization of the ways of working, ensuring digital documentation and comprehensive journal systems [17, 18]. Currently it is mainly welfare technology for digital nightly supervision (cameras) and alarms (passive, sensors, GPS) which are used within the Swedish eldercare sector [19]. While these interventions could be regarded as part of the shift towards working in a more data-driven manner, the health data which gets collected is primarily used for alerting care professionals on emergent incidents rather than prevention and/or early detection, or social care functions. This type of monitoring, or ‘caring from a distance’, is intended to provide older adults with a sense of security while ensuring their independency – in line with how the purpose of welfare technology is defined [6, 17, 19]. Technology that utilizes health data for early detection and/or prevention, such as predictive modelling for fall prevention [20] or AI solutions for assessing the relative risk for disease [13] are two examples of the most recent data-driven solutions put forward. Today, these technologies are not implemented within a Swedish context. However, the increased use of electronic systems for journal documentation, and the integration of multiple systems, enables for these technologies to be utilized [18]. Within Swedish eldercare today, health data is primarily documented in the medical as well as social journal as mandatory for care professionals working within social care for the older adults [21–23]. Recently a new law was introduced which demands that all documentation within eldercare is digitized and made accessible to other care providers [24]. The content of the medical journal (*patientjournal*) abides the same principles as within the general health care sector, and focuses on the planning, execution and evaluation of the medical care interventions and treatments [25]. While person-centered care is recognized as a quality criterion within the medical care plan, integrating the patient’s own perspective into the care plan has been identified as challenging due to its current structure focusing primarily on the medical problems of the patient [26]. The content of the social journal is regulated by the Social Service act; strictly focusing on information relating to the care interventions taken for following up on granted beneficiaries for everyone [21]. Part of the social documentation is also the implementation plan (*genomförandeplan*), which describes goals for planned care interventions, and how these goals are to be realized in more practical terms [27]. The older adult’s own perspectives on his/her care needs are considered [27]. Care interventions responding to the plan should be documented continuously, and revisions of the plan due to changing circumstances is encouraged [27].

### Problematizing data driven health approach in social care

Introducing data-driven health approach within the context of long-term care for older adults could be considered as especially challenging. On a local level, the rationale of a data-driven health approach is to utilize health data as a (knowledge) resource for decision-making within care interventions. While social care is being mentioned as an area in which data-driven health approach could contribute [6, 28], there is a lack of information about exactly in what way this vision could or should be realized. In the policies and industrial vision around data-driven health, the emphasis is clearly on certain types of health data: the kind which describes the objectively measurable aspects of health, often represented in numerical format [28–30]. Previous research has shown that quality of care within long-term care context revolves just as much around how social care is being performed as it is by what kind medical care that is provided [31]. Basing decision mainly on the type of health data which represents the objectively measurable aspects of health could lead to care interventions becoming notably directed towards the medical, rather than the social aspects of care. In using the term medical care, we are referring to the type of care practices which are performed with the intention of curing, restoring or preventing disease; the health data which is utilized within these practices are often biomedical and objectively measurable, computer-documented in a structured way. Social care, on the other hand, incorporates the type of care practices in which care professionals assist older adults in performing daily activities, such as getting dressed, personal hygiene or eating [16]. Social care also incorporates care practices for meeting the social, emotional, cultural and spiritual needs of the residents [31].

Further, studies have shown that the quality of the relationships between caregiver and care receivers is an important determinant for the experience of quality of care for all actors involved in the context of long-term care for older adults [32–39]. In this context, quality of care refers to both quality of medical care and quality of life [38, 39]. As the residents often live permanently in the wards, sometimes for years, the relationships formed within this context are those with which they engage on a daily basis [35]. Hence, these relationships have a determinant effect on the residents overall well-being and sense of belonging [37]. Within the Swedish long-term care context, few studies investigate the perspective of the residents, and results show that older adults emphasize the importance of high quality relationships with the care staff for their experience of care quality in general [40, 41]. Most studies investigate the perspective of the care professionals [42–46]. This body of work highlight how the deprecation of the relational aspects of care have led to negative effects on part of the assistant nurses working within the context of eldercare [43–46]. It also shows that despite the challenges of having to work in an often stressful environment, assistant nurses declare that the relationships which they are able to establish with the older adults as well as their colleagues constitutes what makes them stay and that what gives their work purpose and meaning [42, 43, 46].

The impact that close care provider – care receiver relationships can have on these actors experience of quality of care has received a fair amount of scientific attention. However, what is less explored is the kind of knowledge that is produced within relational care, and how this relates to the quality of care. The emphasis on knowledge is important here: care work produces and requires different kinds of knowledge, which contributes to the quality of care in different ways. Measurable health data such as blood pressure and other vital signs is one such form of knowledge. However, relational care also requires and creates forms of practical knowledge; knowledge which is more difficult to quantify, but has an equally important influence on the experiences of quality of care for the people engaged in the everyday care context. As mentioned, much documentation is already required from the eldercare professionals today. However, most of the relational knowledge falls outside of the scope for what the laws regulations and guidelines that directs documentation allows. With this problematization as a backdrop, we formulated the following research question:

*In what way could the relational aspects of care potentially become affected by the introduction of a data-driven health approach?*

In order to answer this question, we draw on ethnographic material collected at a Swedish long-term care facility. We present our result in the form of propositions which we developed by contrasting our ethnographic insights with the policy discourse around data-driven health approach provided by governmental and industrial stakeholders (presented above).

Previous research adopting a socio-technical perspective have noted that the broader digitalization of health care is changing care practices and caring relations [47, 48]. However, within eldercare much of this research has focused specifically on how technology in the capacity of an embodied tool alters the way that care is organized and practiced, in extension ‘reshaping care interactions and thereby contribute to constituting care in new manners’ [49–52]. While few studies adopting a socio-technical perspective have investigated the use of electronic health records within long-term care for older adults [48], we identify that this perspective needs further exploration within the discussion on health data in general, and in connection to long-term care for older adults in particular.

## Methods

In order to explore how care relationships manifested in practice, and what kinds of knowledge that gets incorporated, we sought to investigate the daily care work at an long-term care facility as it unfolded.

### Methodology

We adopted an ethnographic approach which allows the researcher to observe close-up the day-to-day lives of the people involved in a defined context, with a particular focus on their understanding and interpretation of their experiences [53, 54]. Ethnography has also been upheld as particularly suitable for studying ‘marginalized groups’ – such as older adults residing in long-term care facilities - whose perspectives and experiences are rarely acknowledged [55].

The specific methods adopted were participant observation [53] and informal conversations [56]. All the data retrieved from these methods were documented through taking field notes, using paper and pen. At the start of the fieldwork, we concluded that this was the most suitable way of documenting our observations; using digital tools (smartphone, computer or the like) would have stood out in this environment, as these tools were only used for formal documentation by the assistant nurses. Further, it turned out that the kitchen table – situated in the middle of the ward – constituted an appropriate place for elaborating the short field notes taken while engaging with the participants into more fully written accounts. Included in the field notes were also reflections on certain working documents (formal rules and regulations, steering documents for the care facility in general) that were available at the ward. Thus, all data on which the thematic analysis were conducted was gathered in one corpus: the field notes. The material was analysed iteratively throughout the entire research process adopting theoretically informed thematic analysis (see Data analysis) [57, 58].

### The field: Wind-flower nursing home

The ethnographic fieldwork was conducted in one of the somatic wards at Wind-flower nursing home; a public nursing home situated in the outskirts of the greater Stockholm region, ran by the municipality. It consists of 114 apartments, out of which 64 apartments are intended for residents with somatic care needs, and 50 for those who have been diagnosed with dementia. There are also 12 apartments set aside for short-term residency. All residents have their own apartment equipped with a private bathroom (with a shower) and a bed. Apart from this, the residents are free to furnish their apartments as they wish. The care professionals who work at the nursing home on a daily basis (besides the management) consisted of assistant nurses, care assistants, nurses, doctors, physiotherapists, and occupational therapists. The ethnographic material presented here concerns the practices and experiences of the older adult residents and the assistant nurses.

In order to be appointed an apartment at a nursing home in Sweden today, an individual’s care needs have to be assessed as being so extensive that they cannot be covered by home care [21]. Between the years of 2001 and 2012, the number of beds in Swedish residential care for older adults was reduced by one fourth [59]. Instead home care services have increased; a development which is in line with policies promoting ‘aging-in-place’ [46]. This has led to a situation where those who live in residential care have become older and frailer [46], hence making their care needs more substantial and complex. At the somatic ward where we conducted our field studies, the care needs having to do with the physical limitations of the residents were extensive. Most of the residents were in need of assistance for getting up from their bed in the morning, going to the toilet, getting dressed and taking care of their personal hygiene. This meant that catering to the basic social care needs of the 12 residents at the ward was time intensive. Thus, from around 7 pm to 9am, the assistant nurses were mainly occupied performing care work inside the resident’s individual apartments. The assistant nurses also spent a significant amount of time performing tasks that was not directly connected to social care; tasks which could be placed under the label of ‘house work’. The main part of this work consisted of cleaning (individual apartments as well as common areas), doing laundry, and managing everything regarding “food” (ordering groceries, unpacking groceries, cooking, setting the table, serving food, doing dishes). On top of this, the assistant nurses were responsible for documenting their care work (updating health journals and care plans), keeping track of and planning external visits for the residents (mainly doctor’s visits), as well as coordinating with other parts of the care team (doctor, dentist, nurse and so on).

As mentioned, the assistant nurse’s work incorporates both medical care work and social care work (*omsorg*). However, while they are responsible for performing certain aspects of the medical care work much of the more qualified parts are the liability of nurses and doctors who possess a higher level of expertise and knowledge of medicine and care.

### Fieldwork

The first author (MH) spent 4 months (86 h in total) at the ward, covering both daytime and night-time, weekdays as well as weekends. Participant observation implies that the researcher, to the extent in which it is possible, participate in the daily lives of her interlocutors in a specific context – in this case in the daily lives of the assistant nurses and the residents who worked/lived at the ward [53]. In practice, the emphasis on participation meant that MH participated in the daily care work to the extent that her non-educated care professional role allowed: performing tasks such as serving food to the residents, or giving them a ride in their wheelchairs from one floor to another. What care tasks that were considered suitable to partake in was discussed with the unit chief of the ward before the fieldwork began.

### Data analysis

Data was analysed following Braun & Clark’s [57] ‘six phases’ of thematic analysis. While their step-by-step guidance on thematic analytical process could be interpreted as linear, they emphasize that ‘…it is more recursive process, where movement is back and forth as needed, throughout the phases’ [57]. Hence, the analysis of the ethnographic material took place both during and after the period of the field work; an iterative process in which we moved back and forth between the gathering of data and analysis [58]. Part of this process was to sort out and code prominent analytical themes that occurred in the material; as new ethnographic material was added, this served to either strengthen or challenge the existing categories, adding depth to the analysis as the field work evolved [53, 57]. In accordance with Braun and Clark’s [57] procedure for theoretically driven thematic analysis, we allowed our theoretical position to inform this process: focusing on identifying critical moments where relational care came to the fore. Using previous research (theoretical knowledge) to ‘make sense of the data uncovered in the field research’ is a common approach to theory within ethnography [53, 54]. The analysis departed from an essentialist/realist epistemology combined with a semantic approach [57]. In practice, this meant that the themes were developed based on the explicit statements and practices documented in the field notes, rather than looking for meaning laying beyond the semantic content. Finally, we contrasted our empirical findings with the visions and expectations of a data driven health approach to eldercare coming from industrial and governmental stakeholders; a process which was guided by the main research question ‘*How could the relational aspects of care potentially become affected by introduction of data driven health approach?*’. This resulted in the two propositions presented in the final section of this paper. The two main themes that our thematic analysis resulted in (‘relationships as a source of knowledge’ and ‘relationships as part of quality of life’) are reflected in these propositions. In the ethnographic vignette, as well as the ethnographic excerpts featured in the propositions, we elaborate on these themes and place them in the wider context of a potential data-driven future within eldercare.

### Ethics

Ethical approval was granted by the Swedish Ethical Review Authority (Dnr 2022–03602-01). After gaining approval from the manager of the ward, a group meeting with the assistant nurses working full-time at the ward was scheduled. Research participant information was handed out to the participants a week before the meeting was held. During the meeting, the first author gave an oral presentation of the project, and the participants were encouraged to ask questions. At the end of the meeting, written informed consent was given from all of the assistant nurses.

The process of gaining informed consent from the older adult participants was handled in a different way. Others have noted that ensuring formal consent when doing ethnography among people in a vulnerable position, particularly those suffering from cognitive impairments, can be challenging [55]. Our experience of doing fieldwork at the nursing home resonates with these reflections: while the older adult participating in our study had not received a dementia diagnosis our interactions with them showed that some of them had other types of cognitive impairments, something which the assistant nurses also confirmed. When there is no way of being certain about the participants ability to remember who we were and the reason for us being at the ward questions the value of formal consent gained in the initial stages of the field work [55]. Following Dewing [60, 61], we chose to adhere to the method of ‘process consent’: where consent is seen as an on-going process, something which should be continuously monitored and negotiated along the way. This kind of in situ managing of ethics is part of doing ethnography, particularly when practicing participant observation [62, 63].

The residents living at the ward had not been consulted regarding our presence before we began doing field work. This meant that we were entering into their daily lives and their home without their approval. Some of the older adults showed great interest in our project, and stated that they approved of data being collected about them (both in the form of observations and oral statements which were given to MH). These residents were also the ones who showed a continuous interest in our research, and who seemed to be able to recollect the information about the project which they had received on previous occasion. Others seemed hesitant, or simply uninterested in talking to the MH at all. As mentioned, some displayed problems with remembering the purpose of her stay, frequently asking questions denoting that they though that MH was part of the regular caring staff. Decisions regarding which individual residents to incorporate into the data collection were made based on these observations; the data that this article draws upon only incorporates those residents who appeared to be able to recollect the information about the project that they had received.

The last group that we needed to take into consideration, despite their relatively limited presence in the field, were the relatives and friends of the residents. Before the fieldwork began, an abbreviated and simplified version of the project description was put up on the notice boards at the ward. Besides stressing voluntary participation and anonymity, this document included MH’s email address and phone number, and encouraged anyone who had questions to reach out to her.

Before we move on to present the results, we introduce the ethnographic context with an ethnographic vignette. This vignette is intended to provide the reader with a telling example of what role the relational aspects of care played in this context, and in what way it could be said to connect to the quality of care. Since MH was the one who conducted the fieldwork, reports on ethnographic findings are written in first-person form. Further, all names (participants and places) have been replaced with pseudonyms, in order to ensure full anonymity for all people involved in this study.

### Setting the scene: relational care at Wind-flower nursing home

Room number 9 has just become available, since the man who used to live there has been transferred to the hospital to receive palliative care. He barely managed to settle in before it was time for him to leave, staying at the ward for only a couple of days. I overhear the assistant nurses talk about the new lady (Inga) who is moving into the apartment; transferring from the short-term ward situated one floor above, since the prospects of her moving back to her own apartment are looking bad. I hear them mentioning several medical measures that Inga is in need of: catheter, neck collar, medication every second hour and so on. Based on this conversation, I understand that the assistant nurses are aware of the fact that caring for Inga and catering to her needs will demand a lot of effort and time; time which they do not really have.

Later that day, Anna and Margret (assistant nurses working on the short-term ward) accompany Inga downstairs to help her settle into her new apartment. They come walking through the corridors, pushing Inga in front of them in her permobile. I am standing outside of Inga’s new apartment together with Julia – the head assistant nurse at the ward, who is also Inga’s contact person. Anna and Margret start telling Julia about Inga’s specific care needs; performing a kind of hand-over. They begin with describing Inga’s medical care needs; how the neck collar is to be handled, that she is supposed to wear it 24/7, the medications she is on and how often she is supposed to take them and that she is sensitive to lactose. Sharing this information is done relatively fast – it consists of facts which are important for the assistant nurses at the new ward to know, facts which constitute an important part of what it means to provide Inga good care—but there is not much to discuss or elaborate on in relation to what is being said. They finally state that this information is also available in Inga’s journals. Next, Anna and Margret start telling Julia about who Inga is as a person; her likes and dislikes, which care needs that she has that they have noticed themselves, and what they usually do in order to attempt to meet these needs. Anna, who previously worked at the ward in which I conducted my field studies, first turns to me, then to the rest of the group:

‘…she is very funny this lady, you will like her. She likes to talk, she likes to sing, likes music. She does NOT like to be left alone, then she makes a lot of noise. You will notice that. We usually let her sit in the kitchen with us, and play some music for her. She needs company in order to feel well. She prefers having someone around at all times.’

Julia states that another assistant nurse working at the ward, Elias, used to work with Inga while he was employed within the home help service:

‘…it is good, they know each other. He knows that she used to have both a cat and a dog, which they can talk about.’

As exemplified above, our ethnographic material depicts relational knowledge as a crucial part of what high quality care means in this context. What is important to note is that much of this knowledge is not documented: only that which fits into the regulations regarding what information that the medical and social journal should contain is formally documented. Rather, much of it appears to exist more in the form of ‘informal knowledge’; knowledge which the assistant nurses possess and make great use of, and share orally among each other. It is not considered as health data, since health data by default exists in the form of documented knowledge. What could happen to this practical, most often un-documented relational knowledge in the face of a more data driven health approach? And could this potentially influence the quality of care as experienced by the older adults and assistant nurses? We end this paper by presenting propositions reflecting these questions.

## Results

Viewing our ethnographic material through the observational lense of the problematisation of the policy discourse around data-driven health presented in the introduction, we developed two propositions as a result of the study. In this process, we were guided by our main research question:* In what way could the relational aspects of care potentially become affected by the introduction of a data-driven health approach?*

### Relational knowledge in switching to data-driven health approach

Today, the initial planning of care (*genomförandeplan*) takes place in collaboration between a doctor, a nurse, an assistant nurse (who has been appointed contact person for the resident), the resident and his/her relative(s). The resident’s ‘health journey’, documented throughout his/her previous contacts with different care providers, the social case worker’s need assessment, as well as the resident’s (and his/her relatives) perspective on his/her health are all taken into account. Thus, in these initial meeting (*välkomstsamtal*) all of the actors’ perspectives taken together contribute to creating an individual need-assessment based on a holistic view of the resident’s health.

Over time, I observed how adaptions of the care plan were made—often due to changing circumstances in regards of the residents’ physical health (e.g., deterioration/improvement of disease). Adaptions were also made based on changes in the residents’ own perspective on their needs and goals. On a few occasions, adaptions were also made based on the knowledge that the assistant nurses came to possess by working closely with the residents over an extended period of time: their relational knowledge. This knowledge was constituted by small changes in e.g. mood/behaviour, physical abilities, cognitive capacities, social behaviour, eating patterns and so on. This process is in line with the goals of person-centered care, an approach to care which has been widely implemented within the Swedish context [64]. To a large extent, the assistant nurses’ daily care work is planned and performed based on the care plan, which is constituted by documented health data. Already today, health data thus serves as the basis for providing care customized for the individual resident. However, our ethnographic material depicts a context in which the daily care practices were also shaped by the knowledge which the assistant nurses retrieve from developing and fostering close relationships with the residents.

Being responsible for conducting the social care, and spending by far the most time with the residents, it was the assistant nurses who came to know the residents the most - and established the closest relationships with them. During the first couple of days, I was struck by the level of detail in which the assistant nurses seemed to know the residents. This included knowledge of small details such as what kind of breakfast they preferred (he always eats oatmeal with lingonberry jam and a plain toast); what pets they used to own (they had two bassets named Jasper and Killian); what hobbies they used to engage in (she is a horse girl, she used to compete in show-jumping); were they used to live (she is a true inner-city girl, she has lived her whole life in the city center); the names of the places in which the residents were born (he was brought up in Ljungskile, a small village in on the East coast); their childhood memories (he grew up on a farm, they had horses and cows, they used to harvest the potatoes this time a year) and their family members (her grandson, Max, is an affluent soccer player).

The assistant nurses paid close attention to the residents, and made clear efforts in getting to know them personally. Asking questions about their previous life experiences, about their families, their likes and dislikes, was an integrated part of performing their daily care work: while assisting the residents in e.g. getting dressed for the day, conversations could revolve around how his/her children were doing or whether or not his/her favorite soccer team would be likely to win tonight’s game. Further, I observed how they paid close attention to and made sure to remember seemingly mundane details – such as the temperature of the water someone preferred when showering or if they wanted to have their hair brushed or do it themselves – while performing the daily care practices. The longer the residents stayed on the ward, the more detailed and nuanced the assistant nurses’ knowledge of the residents became. This knowledge was incorporated into the planning of the daily care work and the residents’ daily routines:

- *Olof does not like to stress when having a shower*, something which the assistant nurses took into account when scheduling his shower: knowing that it takes more time than for most of the other residents.
- *For Bodil, it is very important to look nice*: wearing her jewelry and make up, having her hair done with heating coils. The assistant nurses made sure to take this into account when setting their morning-care schedule.
- *Nichole and Amanda prefer having people around*, so the assistant nurses made sure that they were seated in the kitchen while they were preparing the meals. They also noticed that the two of them enjoyed each other’s company; that their relationship seemed to provide them both with a sense of safety and belonging.
- *Saga prefers to drink more coffee than what is usually served* (at breakfast and post-meals), so the assistant nurses made sure that there was always a pot prepared for her to take coffee from.

As our investigation of the policy discourse around data-driven health shows, data-driven health approach focus on using documented health data as a basis for planning/shaping care with an emphasis on the objectively measurable types of data answering to the medical needs of the residents. How to work with social care data within a data-driven health approach is not yet well developed. However, even if this were to be more defined and developed a data-driven health approach is still confined to documented knowledge. Our ethnographic material shows how the oftentimes un-documented relational knowledge helped the assistant nurses to tailor and customize the care in order to fit the individual resident. We suggest that if a more data-driven health approach were to be introduced into this context, relational knowledge risks to become less valued in both the planning of the daily care work and in shaping how the daily care work is performed for the individual older adult.

### Importance of quality of life

Our ethnographic material paints a picture of a context in which the care practices oriented towards improving the residents’ quality of life were just as important for the actors’ experience of quality of care as those which revolved around ensuring a high quality of medical care. For the assistant nurses, this manifested itself through their statements about ‘making things as good as possible for the residents’. Our ethnographic material consists of many statements from the assistant nurses where they explicitly stated that the main reason they were working in long-term care—what they saw as the purpose of their work – was to’make it as good as possible for the residents’. These statements were often told in a manner of frustration and urgency; as if it poured out of a cup that had been on the verge of busting for some time. What seemed to make the assistant nurses so frustrated was that they did not have enough time to dedicate to nurturing their relationships with the residents; that which they saw as the most important part of the job, and that which motivated them to continue doing the job despite a heavy workload (both physically and mentally). ‘Making things as good as possible’ thus constituted a kind of collective working ethos, where everyone seemed to agree that fostering and nurturing close relationships with the residents was the most important part of their job.

As described in the previous section (Relational knowledge in switching to data driven health approach), developing close relationships with the residents was an integrated part of the assistant nurses’ everyday care work. When encountered about her experience of living at the facility, Signe (one of the residents) shared her view on what role relationships played within this context:

‘….well that just the way it is when you get older, its natural…your friends die one after another, and there are fewer and fewer left. All of a sudden you get interest in the death notices, and there are always funerals that you have to attend hehe…here, you are a part of a context, you might not love everyone but you get to know them and they become part of your routine. You feel like you are a part of something.’

I ask her what role the assistant nurses play in relation to her experiences, and she states that:

‘…if you don’t know the person who is taking care of you, then it is hard to feel comfortable and safe. I want to know the person who is entering my room in the morning. The staff who works here, I know them, and they know me. Of course you don’t have the same type of relationship with everyone, but they know me and I know them.’

Our ethnographic material resonates with that which previous research has shown: that within the context of long-term care for older adults, quality of care refers to both quality of medical care as well as quality of life. Relationships contribute to older adults’ socio-emotional well-being, sense of belonging, both important aspects of quality of life [34, 35]. In the policy documents which advocate for implementing a more data-driven health approach within eldercare, quality of care is, to a large extent, equalized with early detection of pathological changes and high precision in the medical interventions taken to prevent, cure or restore disease. Given this, we suggest that implementing a data-driven health approach risk overshadowing the care practices directed towards ensuring a high quality of life for the residents: the relational care practices. This balance between quality of medical care and quality of life could be said to be true for long-term care in general; were care is often more oriented towards alleviating symptoms, rather than curing and/or restoring the residents’ health. That is, when the prospects of curing are no longer an option due to old age, focus is directed towards providing the older adults with the right conditions for leading a life which is as meaningful, dignified and tolerable as possible.

## Discussion

While the introduction of a data-driven health approach has not yet taken place to any extent within the Swedish context of long-term care for older adults, the development within health care in general points towards a situation in which this will become reality. As we have discussed and sought to exemplify by juxtaposing our ethnographic findings with the policies and visions around data-driven health presented by government and industry, this process would benefit from taking account of the context specific aspects found within long-term care for older adults. In every type of health care context, the relationship between caregiver and care receiver constitutes an important component in relation to the quality of care. However, in the context of long-term care for older adults – where patients often live for a longer period of time, and care work consists of just as much social care as medical care – relationships between caregivers and care receivers becomes even more important, and affects the actors experiences of quality of care to an even larger extent.

As the ethnographic excerpts above exemplifies, our material paints a picture of a context in which the relationships between the residents and the assistant nurses played an important role when it came to their experience and assessment of quality of care. In line with findings presented by previous research [42, 43], the assistant nurses described their relationships with the residents as that which gave their work purpose and meaning; an attitude which manifested itself through their collective expression ‘making things as good as possible’. Further echoing previous research [34, 35], the residents described the relationships with the assistant nurses as a prerequisite for feeling safe and comfortable, and as something which contributed to their sense of community and belonging – all important aspects of quality of life. While the specific focus in each of these previous works differs, they all contend that close care provider – care receiver relationships has a crucial influence on these actors’ experiences of quality of care in terms of their socioemotional well-being [32–46]. Thus, since this part of our findings (one out two themes) have been highlighted within previous research they cannot be considered novel as such. However, a specific focus on the knowledge produced within relationships and how this knowledge contributes to quality of care have, to the best of our knowledge, not been investigated within previous research. Our material also highlights how the assistant nurses utilize the intimate and personal knowledge received through engaging in relationships with the residents for tailoring and customizing their care practices in order to fit the individual resident’s needs. Today this knowledge is mainly shared orally and more or less informally among the assistant nurses. The ethnographic vignette constitutes a good example of how this manifested itself in practice: how much of the relational knowledge was used within the emergent caring situations, put to practice in situ without being formally documented. Contrasting these findings with the discourse around data-driven health, we propose that this undocumented knowledge risk becoming less valued in the face of a data-driven health approach which utilizes documented knowledge as its basis for decision-making around care interventions. These findings could also be placed within a broader discussion around digitalization or ‘datafication’ [1] of health care in general; a topic which has received a fair amount of social scientific scholarly attention recently (see Background). Since research which focus specifically on the context(s) of eldercare is lacking within this body of work, this strengthens our assertion that our study contributes to filling a knowledge gap which deserves further scholarly attention.

In Sweden, the Covid-19 pandemic brought two major concerns within long-term care for older adults to the surface: the lack of medical expertise and the insufficient competence level among assistant nurses and care assistants [65]. Since then, significant amounts of state funds have been dedicated to improving these areas [66], and much of the public debate around eldercare have come to revolve around these areas as well. As Nakrem et al. [39] notes, there needs to be a balance between medical, physical and psychosocial care in residential care for older adults. Notwithstanding the importance of heightening the level of medical expertise or occupational competence among assistant nurses and care assistants, the importance of relational aspects for quality of care tends to be forgotten within these recent public discussions. As mentioned, there already exists a fair amount of research that have investigated the role that relationships play within care for older adults [32–46]. However, the majority of these studies were conducted around a decade ago. Our study goes beyond the currently dominating focus on lack of medical expertise and insufficient competence levels, but more up-to-date studies investigating the relational aspects are needed to serves this purpose.

This paper highlights in which ways the introduction of a data-driven health approach might affect the care that is currently being practiced, with a particular focus on those relational aspects of care that have proven to be determinant for the older adults and care professionals (assistant nurses) perceptions of the quality of care which is performed. Our findings are therefore in support of previous research departing from a socio-technical perspective that the introduction of new technology has the capacity to change already existing care practices in a profound way [48–52]. Albeit, these previous studies also constitutes an example of another common tendency within research on new technologies within health care; due to its focus on ‘embodied tools’ interacting with social environments, data collection often takes place either at the stage of introduction, implementation or sometimes during the pilot study phase [48–52]. Since data-driven health approach within long-term care for older adults currently exists more on a visionary stage than as part of an actual every day care reality, conducting research on this topic *before* its introduction can contribute with findings that could serve as a basis for planning and designing future data-driven health interventions. In other words; rather than waiting for the introduction of data-driven health approach to take place in this context, scholarly efforts ought to be directed towards doing research which could help this (potential) future developments to unfold in a way which is in alignment with the everyday care realities in which they are to be introduced. Our current study (Hellstrand et al. forthcoming), in which we aim to gain better understanding of how well the health data currently being collected about an older adult corresponds with their own perspective of their health and well-being, represents our latest attempt at doing this. We encourage others to follow.

### Limitations

Due to its small sample size and case-like design, ethnography has been criticised for its lack of generalizability [53]. The fact that our study only reports findings from one single site, combined with the relatively low number of participants (12 older adults and 8 assistant nurses) may be considered as a limitation. With that said, we would like to point out that our aim with the present study was to gain an in-depth understanding of how the participants came to understand and interpret their experiences of engaging in care relations. Gaining this kind of understanding demands both sufficient time spent in the field, as well as developing field relations built on trust and respect. This last part is particularly relevant when it comes to conducting field studies in peoples’ most intimate sphere – their home – which was the case for the residents participating in our study. Observations, participation in daily activities combined with informal conversations allow the researcher to immerse into the field, subsequently getting to know the participants and notice what is meaningful to them and how it manifest itself both within their actions and within statements. For this project (part of MH’s PhD-project), a limited amount of time was set for conducting fieldwork (4 months). Thus, we concluded that focusing on a single site would provide a higher quality of data than what we would have gained from spreading out time visiting multiple field sites. While we do not assert that our result are generalizable to a finite population, we believe that they can be valuable as a point of departure for stakeholders who wish to implement data-driven health approach in a way which adheres to older adults’ and assistant nurses’ perspectives and experiences.

## Conclusion

The challenges for introducing a more data-driven health approach into the context of long-term care for older adults which we have identified could be summarized in the following way: if a clearer focus, and stronger emphasis, is placed on the documented aspects of care, this could lead to a situation in which important benefits of utilizing the relational knowledge gets dismissed. This concerns both the on-going adoption of the older adults’ care plans, as well as the way that the assistant nurses value this type of knowledge, and the extent in which they incorporate it into their daily work. Further, if emphasis is more on objectively measurable and documented health data quality of care risk becoming more focused on quality of the medical care than on the quality of life. That is, if the rationale for practicing high quality care states that care should be both planned and shaped based on objectively measurable and documented health data there is a risk that the assistant nurses focus more on abiding these principles than on investing time and effort into practicing relational care contributing to the residents’ quality of life. In sum, our results highlight the importance of taking the relational aspects of care into consideration, both during the planning and implementation phase for data-driven health approach within the context of long-term care for older adults. Doing this could result in data-driven health approach supporting the knowledge and expertise which the actors already possess, as well as contributing to heightening the quality of the medical care without compromising quality of life.

## Data Availability

All empirical data (field notes and transcriptions) are stored on a secure server at KTH Royal Institute of Technology. Due to ethical concerns, the material is not publicly available. An anonymized version of empirical material analysed in the current study are available upon reasonable request from the corresponding author. Please note that the material is in Swedish.
